# Loss of long-term non-progressor and HIV controller status over time in the French Hospital Database on HIV - ANRS CO4

**DOI:** 10.1371/journal.pone.0184441

**Published:** 2017-10-02

**Authors:** Sophie Grabar, Hana Selinger-Leneman, Sophie Abgrall, Gilles Pialoux, Laurence Weiss, Dominique Costagliola

**Affiliations:** 1 Sorbonne Universités, INSERM, UPMC Univ Paris 06, Institut Pierre Louis d’épidémiologie et de Santé Publique (IPLESP UMRS 1136), F75013, Paris, France; 2 Assistance Publique Hôpitaux de Paris (AP-HP), Groupe hospitalier Cochin Broca Hôtel-Dieu, Unité de Biostatistique et d’épidémiologie, Paris, France; 3 Université Paris Descartes, Sorbonne Paris Cité, Paris, France; 4 APHP, Hôpital Béclère, Clamart, France; 5 Faculté de Médecine Paris Sud, Le Kremlin Bicêtre, France; INSERM U1018, Centre de recherche en Epidémiologie et Santé des Populations, Université Paris Sud, Le Kremlin Bicêtre, France; 6 APHP, Hôpital Tenon, Paris, France; 7 UPMC Univ Paris 06, France; 8 APHP, Hôpital Européen Georges Pompidou, Paris, France; University of Pittsburgh Centre for Vaccine Research, UNITED STATES

## Abstract

**Objectives:**

We studied the frequency and risk factors for loss of long-term non-progressor (LTNP) and HIV controller (HIC) status among patients identified as such in 2005 in the French Hospital Database on HIV (FHDH-ANRS CO4).

**Methods:**

We selected patients who were treatment-naïve and asymptomatic in 2005 (baseline). Those with ≥8 years of known HIV infection and a CD4 cell nadir ≥500/mm^3^ were classified as LTNP and those with ≥10 years of known HIV infection and 90% of plasma viral load (VL) values ≤500 copies/ml in the absence of cART as HIC. cART initiation without loss of status and death from non AIDS-defining causes were considered as competing events.

**Results:**

After 5 years of follow-up, 33% (95%CI; 27–42) of 171 LTNP patients and 17% (95%CI; 10–30) of 72 HIC patients had lost their status. In multivariable analyses, loss of LTNP status was associated with lower baseline CD4 cell counts and CD4/CD8 ratios. Only VL was significantly associated with loss of HIC status after adjustment for the baseline CD4 cell count, the CD4/CD8 ratio, and concomitant LTNP status. The hazard ratio for loss of HIC status was 5.5 (95%CI, 1.5–20.1) for baseline VL 50–500 *vs* ≤50 cp/mL, after adjustment for the baseline CD4 cell count.

**Conclusions:**

One-third of LTNP and one-fifth of HIC patients lost their status after 5 years of follow-up, raising questions as to the possible benefits and timing of ART initiation in these populations.

## Introduction

Individuals who spontaneously control HIV infection provide a model of natural protection relevant to vaccine development and immune intervention. Although many definitions coexist [1], these patients are generally defined as either long-term non progressors (LTNP) or HIV controllers (HIC), depending on whether they are defined based on immunologic or virologic factors and on protection of AIDS-defining events in the absence of antiretroviral treatment. Few studies have focused on the long-term outcomes of such rare patients [2–9], who will become more difficult to identify as new treatment guidelines recommend treatment initiation as early as possible. Therefore the management of the individuals already identified with such status is an issue for both patients and caregivers [10].

Here we examined the frequency and predictive factors for loss of LTNP and HIC status over time among patients identified in the French Hospital Database on HIV (FHDH_ANRS CO4) in 2005 [11].

## Patients and methods

### Patients

The French Hospital Database on HIV (FHDH, ANRS CO4) is a nationwide hospital-based cohort created in 1989, in which clinical and biological data on HIV-infected patients throughout France are prospectively recorded [12]. To date more than 140 000 patients have been included and signed an informed consent. Data submitted by the participating centres to the data coordinating and analysis centre are anonymized, then encrypted. The FHDH was approved by the institutional ethic committees, Commission Nationale de l’Informatique et des Libertés (CNIL) on 27 November 1991 (Journal Officiel, 17 January 1992). Using the same definition as previously [11] we characterize two groups of HIV1-infected adults who attended an FHDH follow-up visit in 2005 while asymptomatic and antiretroviral-naïve, and who had had at least three CD4 cell counts measurements during the previous 5 years. Long-term non-progressors (LTNP) were defined as patients known to have been HIV1-seropositive for at least 8 years and who had a CD4 nadir ≥500 cells/mm^3^. HIV controllers (HIC) were patients known to have been HIV1 seropositive for more than 10 years and in whom 90% of plasma HIV RNA values were below 500 copies/ml. Only patients who had at least one follow-up visit after 2005, with CD4 and plasma HIV RNA measurements, were eligible for this analysis.

### Methods

We used a competing-risk approach to estimate the cumulative incidence of loss of LTNP and HIC status [13], as defined by progression to AIDS or death from an AIDS-defining cause, or as two consecutive CD4 cell counts <500/mm^3^ in the LTNP group and more than 10% of HIV RNA values >500 cp/mL in the HIC group. ART initiation without loss of status and death from non AIDS-defining causes, were considered as competing events.

Follow-up was measured from the date of status identification in 2005 (baseline) until loss of status, a competing event, or the last follow-up visit, whichever occurred first. The subdistribution hazard ratio (sdHR), hereinafter referred as”risk”, for status loss was estimated, along with its 95% confidence interval (95%CI)[13]. Multivariable analyses were adjusted for baseline characteristics with p values below 0.1 in univariable analyses. Backward selection retained only those variables with p values below 0.1. For the HIC group, only two variables could be entered in the multivariable model, owing to an insufficient number of events. For continuous variables, the choice of metric (categorized or continuous) was based on the lowest value of Akaike's criterion (AIC).

SAS software version 9.4 (SAS Institute, Cary, NC) was used for all analyses. P values <0.05 were considered to denote statistical significance.

## Results

In 2005, among the 202 LTNP patients included in our previous analysis [11], 184 patients (91%) still had LTNP status: an update showed that 12 patients started antiretroviral treatment before 2006, 4 patients had developed AIDS, and data were missing for 2 patients. Among these 184 LTNP patients, 171 had a follow-up visit after 2005 and were thus included in this analysis. Of them 22 had a CD4 cell count nadir of at least 600/ml, and a positive CD4 cell slope and were previously referred as “Elite LTNP” [11].

Among the 101 previously studied HIC patients, an update showed that 18 patients started antiretroviral treatment before 2006, one patient developed AIDS, and two patients were also infected by HIV-2. Among the remaining 80 HIC patients, 72 had a follow-up visit after 2005 and were thus included in this analysis. Of them 50 had a last HIV RNA value below 50 cp/mL and were previously referred as “Elite HIC” [11]. Thirty-five patients had both LTNP and HIC status.

The patients’ baseline characteristics are described in **Table 1**. The mean follow-up after 2005 was 4.85 (+/- 2) years.

**Table 1:** Characteristics of LTNP and HIC patients identified in 2005 who maintained or lost their status during follow-up.

	LTNP in 2005	Still LTNP during follow-up	Lost LTNP status during follow-up	HIC in 2005	Still HIC during follow-up	Lost HIC status during follow-up
Total N	N = 171	N = 100	N = 55	N = 72	N = 52	N = 12
**Sex** n(%)						
Women	59 (34.5)	36 (36.0)	17 (30.9)	26 (36.1)	20 (38.5)	3 (25)
Men	112 (65.5)	64 (64.0)	38 (69.1)	46 (63.9)	32(61.5)	9 (75)
**Transmission group** n (%)						
Men who have sex with men	65 (38)	38 (38.0)	23 (41.8)	7 (9.7)	6 (11.5)	1 (8.3)
IVDU	37 (21.6)	21 (21.0)	11 (20.0)	31 (43.1)	23 (44.2)	4 (33.3)
Heterosexual	56 (32.7)	32 (32.0)	19 (34.5)	19 (26.4)	16 (26.9)	4 (33.3)
Others	13 (7.6)	9 (9.0)	2 (3.6)	15 (20.8)	9 (17.3)	3 (25)
**Geographic Origin** n(%)						
France	152 (88.9)	92 (92.0)	48 (87.3)	68 (94.4)	48 (92.3)	12 (100)
Sub-Saharan Africa	6 (3.5)	5 (5.0)	1 (1.8)	3 (4.2)	3 (5.8)	
Other	13 (7.6)	3 (3.0)	6 (10.9)	1 (1.4)	1 (1.9)	
**Year of HIV diagnosis** median (IQR)	1992 (1988–1995)	1992 (1988–1995)	1992 (1989–1996)	1988 (1986–1992)	1988 (1986–1992)	1988 (1987–1990)
**Hepatitis B** antigen positive	9 (5.3)	8 (8.0)	1 (1.8)	6 (8.3)	5 (9.6)	0 (0)
**Hepatitis C** antibody positive	39 (22.8)	22 (22.0)	13 (23.6)	37 (51.4)	24 (46.2)	7 (58.3)
**Age in 2005** median (IQR)	42 (39–48)	42 (40–47)	41 (38–47)	44 (41–52)	44 (40–49)	45 (43–53)
**CD4 cell count (/mm**^**3**^**)** in 2005 Median (IQR)	778 (644–939)	862 (700–980)	668 (568–773)	721 (547–946)	771 (578–1015)	570 (429–671)
**CD4 cell nadir in 2005 (/mm**^**3**^**)** median (IQR)	610 (550–703)	635 (552–761)	568 (537–624)	504 (334–665)	564 (387–700)	570 (429–671)
**pVL (copies/ml) in 2005** median (IQR)	1961 (140–13000)	500 (50–3721)	10300 (1820–37600)	50 (50–122)	50 (50–50)	260 (100–437)
**CD8 cell count in 2005** median (IQR)	1050 (774–1482)	909 (698–1263)	1309 (997–1717)	756 (616–1119)	817 (647–1196)	752 (598–957)
**CD4/CD8 in 2005** n (%)						
**<1**	110 (64.3)	56 (56.0)	44 (80.0)	36 (50)	24 (46.2)	6 (50)
**> = 1**	35 (20.5)	29 (29.0)	1 (1.8)	31 (43.1)	23 (44.2)	6 (50)
**Missing**	26 (15.2)	15 (15.0)	10 (18.2)	5 (6.9)	5 (9.6)	
**Nb of biological measurements from characterization in 2005 until** last follow-up. Median (IQR)	12 (6–18)	9 (5–13)	17 (12–26)	10 (6–17)	9 (5–14)	16 (11–24)
**Interval between measurements (months)**	5(4–7)	6 (4–9)	4 (3–6)	6(3–8)	6 (4–9)	5 (4–7)

IDVU: intravenous drug user IQR: interquartile range; pVL: plasma HIV RNA

### Long-term non-progressors

**Fig 1:**
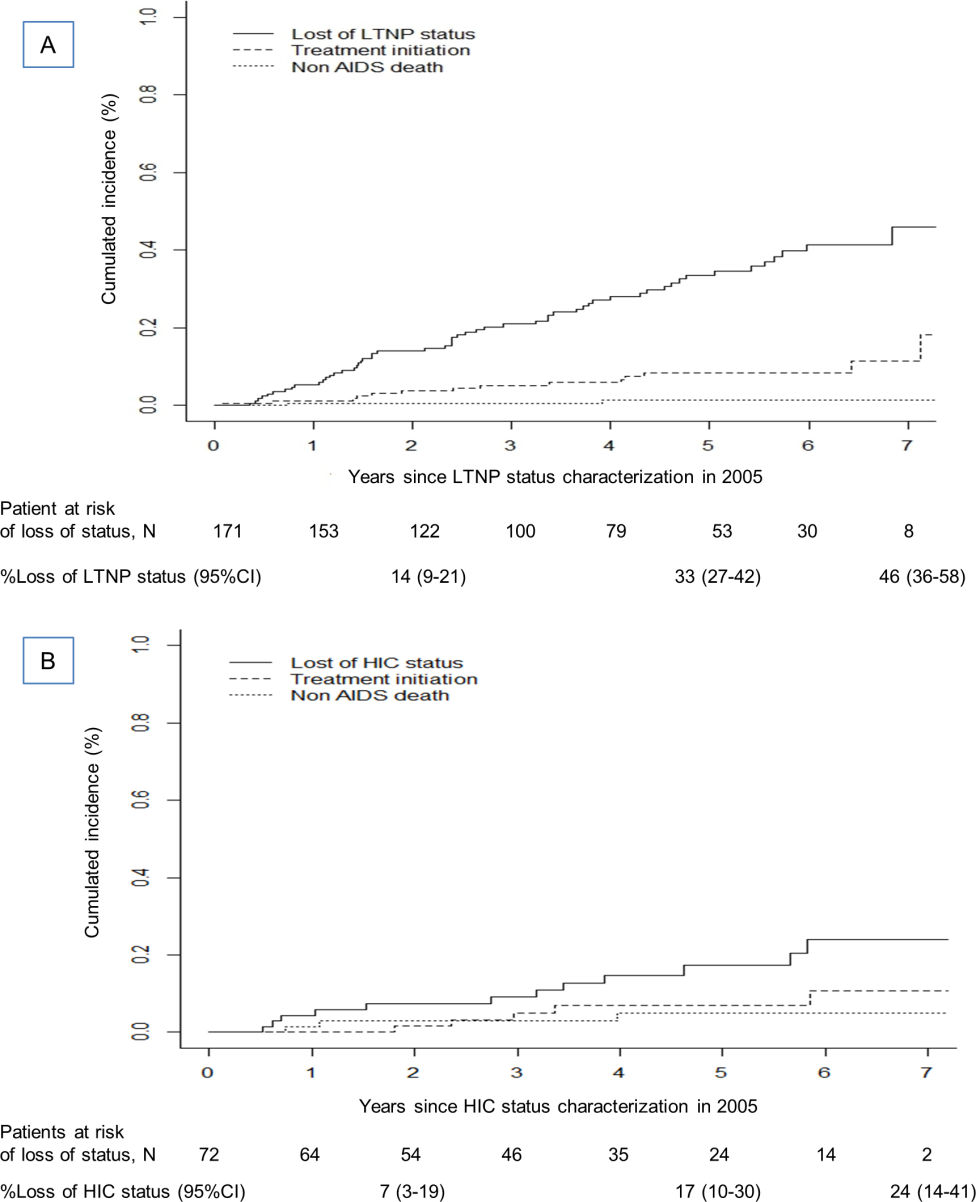
**Cumulative incidence of loss of LTNP (panel A) and HIV controller (panel B) status, treatment initiation and non AIDS deaths since 2005. A:** Long-term non-progressors (n = 171). **B:** HIV controllers (n = 72).

55 of the 171 LTNP patients lost their status: one developed AIDS (herpes simplex chronic ulcer, or pneumonitis, or esophagitis), 14 patients started cART without loss of status, and 2 patients died of non AIDS-defining causes (alcoholic cirrhosis, and hepatocellular carcinoma in an HCV-coinfected patient). Two patients with both LTNP and HIC status lost their LTNP status. **Fig 1A** shows the cumulative incidence of loss of LTNP status during up to 7 years of follow-up. At 5 years, the risk of loss of LTNP status was 0.33 (95%CI, 0.27–0.42).

In univariable analyses, baseline factors associated with LTNP status loss were a low CD4 cell count, a low CD4/CD8 ratio (documented in 145 patients, of whom 45 lost their LTNP status), high HIV RNA load, and no concomitant HIC status **(Table 2)**.

**Table 2:** Univariable and multivariable analyzes for loss of LTNP and HIV controller status.

	LTNP univariable analyze (n = 171)				LTNP Multivariable analyze (n = 145)				HIC Univariable analyze (n = 72)			
	sdHR	95%CI		p	asdHR	95%CI		p	sdHR	95%CI		p
**Sex**												
M	1								1			
F	0.92	(0.52	1.65)	0.7814					0.51	(0.14	1.86)	0.31
**Transmission group**				0.6519								0.87
Heterosexual	1								1			
MSM	1.14	(0.62	2.09)						1.21	(0.15	9.88)	
IDVU	0.89	(0.42	1.86)						0.69	(0.18	2.62)	
Others	0.46	(0.11	2.11)						1.26	(0.29	5.46)	
**Age in 2005 (by 10 yrs)**	0.87	(0.60	1.26)	0.4713					1.16	(0.73	1.83)	0.53
**CD4 cell count in 2005 (log2)**	0.10	(0.04	0.22)	0.0000	0.31	(0.10	0.94)	0.04	0.47	(0.22	1.02)	0.06
**CD4/CD8 ratio in 2005**	0.68	(0.63	0.77)	0.0000	0.75	(0.63	0.86)	< .0001	0.94	(0.85	1.05)	0.29
(/0.1 increase)												
**Viral Loal in 2005**												
log10	1.89	(1.45	2.47)	0.0000	1.30	(0.82	2.07)	0.27				
< = 50									1			
]50–500]									7.14	(1.99	25.57)	0.003
**HIC status**												
No	1											
Yes	0.11	(0.03	0.44)	0.0015	0.74	(0.17	3.27)	0.70				
**LTNP status**												
no									1			
yes									0.30	(0.09	1.01)	0.0051

IDVU: intravenous drug user; MSM Men who have sex with men; sdHR: sub-distribution Hazard Ratio; asdHR: adjusted sub-distribution Hazard Ratio

Being an elite LTNP was associated with a reduced risk of LTNP loss although not significantly (sdHR 0.37, 95%CI 0.12–1.18; p = 0.09). In the final multivariable model, the baseline CD4 cell count and CD4/CD8 ratio remained significantly associated with loss of LTNP status after adjustment for baseline HIV RNA load. A 2-fold increase in the baseline CD4 cell count was associated with a 69% lower risk of LTNP status loss (sdHR 0.31, 95%CI 0.10–0.94). The CD4/CD8 ratio was inversely associated with status loss (per 0.1 increase in the CD4/CD8 ratio: sdHR 0.75, 95%CI 0.63–0.86). Baseline HIV RNA was not significantly associated with LTNP status loss (sdHR 1.30, 95%CI 0.82–2.07 p = 0.27). Of note, CD4/CD8 ratio was slightly higher in elite LTNP than in non-elite LTNP; median 0.78 (IQR 0.67–1.19) *vs* 0.59 (IQR 0.3–0.97) respectively.

### HIV controllers

Among the 72 HIC, 12 patients lost HIC status by losing HIV RNA control, 5 patients initiated cART without status loss and 3 died from non AIDS cause (alcoholic cirrhosis, hepatocellular carcinoma, and stomach cancer). Among the patients who maintained their HIC status, 40% of patients had a CD4 nadir below 500/mm^3^ at baseline and 21% a CD4 nadir below 350/mm^3^. **Fig 1B** shows the cumulative incidence of loss of HIC status. At 5 years, the risk of HIC status loss was 0.17 (95%CI, 0.10–0.30). At the end of follow-up, 37/72 patients (51%) had HIV RNA values below 50 copies/mL.

In univariable analyses, baseline HIV RNA load was significantly associated with HIC status loss (sdHR 7.14, 95%CI 1.99–25.57 for values between 50–500 vs ≤50 cp/mL ie elite HIC). Low CD4 cell counts and concomitant LTNP status both had borderline significance (p = 0.06), while the CD4/CD8 ratio was not significant (p = 0.29). When adjusted for baseline HIV RNA (not shown), neither the CD4 cell count (sdHR 0.54, 95%CI, 0.22–1.30; p = 0.17) nor concomitant LTNP status (sdHR 0.45, 95%CI, 0.13–1.55; p = 0.20) was significantly associated with loss of HIC status, while HIV RNA load remained significant, with an sdHR above 5.5 for VL 50–500 vs ≤50 cp/mL.

## Discussion

In this study of a large hospital cohort, we found that 33% of long-term non-progressors and 17% of HIV controllers had lost their status 5 years after being identified as such. Interestingly, in addition to a lower baseline CD4 cell count, a lower CD4/CD8 ratio was associated with loss of LTNP status, whereas viral load >50 cp/ml was the strongest predictor of loss of HIC status.

### Strengths and limits

Few studies have examined the long-term outcome of LTNP and HIC patients [2–9]. Contrary to previous studies, we used competing-risk analysis to accurately estimate the risk of status loss while avoiding overestimation. One limitation is that, despite the large size of the cohort, there were too few events in the HIC population for a complete and powerful multivariable analysis. The lack of data on HIV-DNA load is also a limitation, as HIV-DNA levels, which are known to be low in HIC patients [14], have been identified as a determinant of disease progression, in addition to the CD4 cell nadir and HIV-RNA [3].

### Associated factors

One third of our LTNP patients lost their LTNP status after 5 years. Loss of LTNP status was associated with a lower baseline CD4/CD8 ratio and a lower CD4 cell count, whereas HIV RNA load was the only factor associated with loss of HIC status. HIC patients with HIV RNA values between 50 and 500 cp/mL had a much (5.5-fold) higher risk of progression than patients with values below 50 cp/mL (ie elite HIC). The CD4/CD8 ratio, concomitant LTNP+HIC status and the CD4 cell count did not reach statistical significance. However, the sdHR for the CD4/CD8 ratio was close to 1, clearly indicating no association with loss of HIC status. By contrast, the sdHRs for the CD4 cell count and concomitant LTNP+HIC status were both close to 0.5, and the lack of statistical significance may have resulted from a lack of power.

### Implications of the results

It is important to identify factors associated with loss of LTNP and HIC status, because the few relevant reports suggest that ART initiation before loss of status might be beneficial in these populations. Indeed, Hatano et al. [15] found that ART initiation in HIC patients reduced inflammatory marker levels, while Boufassa et al. [16] noted an increase in the CD4 cell count on treatment (albeit smaller than in other patients), thus supporting ART initiation before the CD4 cell count decline below 500/mm^3^.

Given the relatively frequent loss of LTNP status observed here, our data suggest that all LTNP patients should begin cART without delay. This is in line with the results of the INSIGHT-START trial, which showed a clear clinical benefit of immediate *vs* deferred ART in asymptomatic adults with CD4 cell counts above 500/mm^3^ [17], especially in those with high plasma HIV RNA and a CD4/CD8 ratio below 0.5 [18].

Treatment of HIC patients with stable immunovirologic status is controversial [10]. Some argue that treatment could help to control persistent inflammation and thereby prevent clinical events. Recently, Crowell et al. [19] found that elite controllers were hospitalized twice as often as patients on effective cART, mainly for cardiovascular disorders compatible with excess inflammation. Likewise, Pereyra et al. [20] reported that elite controllers had increased coronary and immune activation. By contrast, in younger population (median age 27 years) such higher rate of cardiovascular hospitalization rates were not observed [21]. We found no association between the baseline CD4/CD8 ratio and loss of HIC status, whereas HIV RNA values 50–500 cp/mL were associated with a 5.5-fold higher risk of progression compared to ≤50 cp/mL. Thus, HIC patients with HIV RNA values above 50 cp/mL and/or CD4 cell counts below 500/mm^3^ should be offered cART, especially as 1 in 5 of such patients will lose their HIC status after 5 years. Regarding HIC patients with HIV RNA values below 50 cp/mL and CD4 cell counts above 500/mm^3^, the answer to whether or not cART should be started is less clear. However if cART were to be deferred, the stability of the CD4 cell count should be closely monitored. If cART were to be initiated, the timing of treatment initiation should be considered with care, and the reasons must be clearly explained, as these patients have learn to cope with their exceptional status [22] and some may have come to believe that they would not require treatment. In these patients treatment initiation will disrupt their disease’s trajectory [22].

## Conclusions

Our results indicate that one-third of LTNP patients and one-fifth of HIC patients will lose their status within 5 years, and highlight the question of ART initiation in these patients. However, whatever the status, LTNP or HIC, initiation of ART should be carefully discussed with the patients in order to find the adequate time to interrupt a long period of exceptionality.
